# Spatially coherent and topographically organized pathways of the human globus pallidus

**DOI:** 10.1002/hbm.25147

**Published:** 2020-08-05

**Authors:** Salvatore Bertino, Gianpaolo Antonio Basile, Alessia Bramanti, Giuseppe Pio Anastasi, Angelo Quartarone, Demetrio Milardi, Alberto Cacciola

**Affiliations:** ^1^ Brain Mapping Lab, Department of Biomedical, Dental Sciences and Morphological and Functional Images University of Messina Messina Italy; ^2^ IRCCS Centro Neurolesi “Bonino Pulejo” Messina Italy

**Keywords:** basal ganglia, movement disorders, neuroimaging, parcellation, structural connectivity, tractography

## Abstract

Internal and external segments of globus pallidus (GP) exert different functions in basal ganglia circuitry, despite their main connectional systems share the same topographical organization, delineating limbic, associative, and sensorimotor territories. The identification of internal GP sensorimotor territory has therapeutic implications in functional neurosurgery settings. This study is aimed at assessing the spatial coherence of striatopallidal, subthalamopallidal, and pallidothalamic pathways by using tractography‐derived connectivity‐based parcellation (CBP) on high quality diffusion MRI data of 100 unrelated healthy subjects from the Human Connectome Project. A two‐stage hypothesis‐driven CBP approach has been carried out on the internal and external GP. Dice coefficient between functionally homologous pairs of pallidal maps has been computed. In addition, reproducibility of parcellation according to different pathways of interest has been investigated, as well as spatial relations between connectivity maps and existing optimal stimulation points for dystonic patients. The spatial organization of connectivity clusters revealed anterior limbic, intermediate associative and posterior sensorimotor maps within both internal and external GP. Dice coefficients showed high degree of coherence between functionally similar maps derived from the different bundles of interest. Sensorimotor maps derived from the subthalamopallidal pathway resulted to be the nearest to known optimal pallidal stimulation sites for dystonic patients. Our findings suggest that functionally homologous afferent and efferent connections may share similar spatial territory within the GP and that subcortical pallidal connectional systems may have distinct implications in the treatment of movement disorders.

## Graphical abstract

The identification of internal globus pallidus (GP) sensorimotor territory has therapeutic implications in functional neurosurgery settings. This study is aimed at assessing the spatial coherence of striatopallidal, subthalamopallidal and pallidothalamic pathways by using tractography‐derived connectivity‐based parcellation. Our results suggest that the general topographical organization of connectivity parcels is highly consistent across the internal and external GP, regardless the bundle of interest, corroborating the hypothesis that different pathways, running parallel and in series to one other, may share the same spatial organization pattern. Indeed, we found high similarity among functionally homologous connectivity maps derived from the striatopallidal, subthalamopallidal and pallidothalamic tracts. In other words, we show neuroimaging evidences suggesting the presence of partially segregated yet integrated functional territories within the GP, derived from connectivity patterns that maintain a consistent topographical arrangement throughout the entire cortico‐basal ganglia‐thalamo‐cortical circuitry.

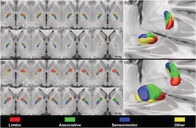

## 1 INTRODUCTION

The globus pallidus (GP) is subdivided into an internal (GPi) and an external (GPe) segment, each subserving different functions in the basal ganglia system (Yelnik, 2002). While both the GPe and GPi receive main afferent fibers from the striatum and subthalamic nucleus (STN), their efferent connectivity patterns show some diversities. Indeed, the GPe mainly projects to the STN, while the GPi chiefly projects to the thalamus (Nambu, 2007; Parent & Parent, 2004).

The most accepted model of basal ganglia functional anatomy posits that cortico‐basal ganglia circuits are topographically organized in parallel loops, each involving distinct territories of striatum, GP, substantia nigra, STN, and thalamus. According to this model, connections from distinct cortical areas are likely to be topographically organized in segregated, identifiable, yet integrated territories in the whole basal ganglia system (Alexander, Crutcher, & Delong, 1990; Cacciola, Milardi, Anastasi, et al., 2016; Cacciola, Milardi, Anastasi, & Quartarone, 2018; Cacciola, Milardi, & Quartarone, 2016; Grabli et al., 2004; Karachi et al., 2002, 2005; Milardi et al., 2019; Quartarone et al., 2019; Saga, Hoshi, & Tremblay, 2017).

In the last decades, this model has extensively influenced stereotactic and functional neurosurgery. The identification of putative functional territories within the designed target, indeed, is of pivotal importance to improve clinical outcomes and avoid collateral effects, which are supposed to be related to stimulation/ablation of inappropriate territories (Dembek et al., 2019; Reich et al., 2019).

GPi implantation for deep brain stimulation (DBS) is a well‐established therapeutic approach in dystonic patients, as well as in drug‐refractory Parkinson's disease (PD) and Gilles De la Tourette syndrome (Deuschl et al., 2006; Weaver et al., 2012). On the other hand, the GPe has been proposed as an effective target for DBS in both primary and secondary dystonia (Houeto et al., 2007; Vitek, Zhang, Hashimoto, Russo, & Baker, 2012), as well as for severe insomnia in PD patients (Castillo et al., 2020).

Recently, DBS has allowed for the electrophysiological characterization of basal ganglia in movement disorders, identifying disease‐specific oscillatory patterns (Silberstein et al., 2003). In particular, studies conducted on implanted dystonic patients showed that: (a) pallidal theta oscillatory activity, a recently proposed dystonia physiomarker, was significantly correlated with severity of symptoms and (b) stimulation delivered in proximity of the theta peak amplitude provided the best outcomes, in terms of motor improvement, both at short and long term. Moreover, electrophysiological mapping revealed that the site of peak of theta activity was close to coordinates of optimal stimulation sites for dystonia, apparently located in the sensorimotor territory of the GPi (Neumann et al., 2017; Scheller et al., 2019).

Diffusion MRI and tractography (Beckmann, Johansen‐Berg, & Rushworth, 2009; Saygin et al., 2015; Saygin, Osher, Augustinack, Fischl, & Gabrieli, 2011) have been used to disentangle the topographical organization of the main white matter bundles connecting basal ganglia structures (Cacciola, Milardi, Bertino, et al., 2019; da Silva et al., 2017; Lehéricy et al., 2004; Lenglet et al., 2012; Middlebrooks, Tuna, Grewal, et al., 2018; Patriat et al., 2018; Plantinga et al., 2018). In particular, structural connectivity‐based parcellation (CBP) (Eickhoff, Thirion, Varoquaux, & Bzdok, 2015) has been employed to reconstruct different spatial maps of GP, each disclosing complementary facets of its topographical organization (Cacciola, Milardi, Bertino, et al., 2019; da Silva et al., 2017; Ewert et al., 2018). Preliminary studies demonstrated the feasibility of such approach to identify the sensorimotor territory of GPi in functional neurosurgery contexts (Middlebrooks, Tuna, Grewal, et al., 2018; Patriat et al., 2018). However, consistent methodological differences among various works, together with their small sample size, strongly affect the generalization of such results. Therefore, the lack of a comprehensive, unified framework of GP connectivity and functional topography limits the possible translation of such approaches in a clinical context (Middlebrooks, Tuna, Grewal, et al., 2018; Sweet, Pace, Girgis, & Miller, 2016; Tisch et al., 2007).

In the present study, aimed at characterizing the spatial, topographical arrangement of subcortical connectivity patterns within the GPi and GPe, we combined multi‐shell multi‐tissue constrained spherical deconvolution (MSMT‐CSD) (Jeurissen, Tournier, Dhollander, Connelly, & Sijbers, 2014; Tournier, Calamante, & Connelly, 2012) tractography with a two‐stage hypothesis‐driven CBP approach on high quality 3T diffusion MRI data of 100 unrelated healthy subjects from the Human Connectome Project (HCP) repository (Behrens et al., 2003). For each GP segment, distinct connectivity maps from striatopallidal, subthalamopallidal, and pallidothalamic pathways were reconstructed and the spatial consistency among functionally homolog maps was assessed by means of Dice coefficient. In addition, we evaluated the reproducibility of the parcellation procedure by calculating intersubject similarity measures (da Silva et al., 2017; Traynor et al., 2010). Finally, we investigated the spatial relationship between putative sensorimotor maps obtained from different bundles of interest and publicly available coordinates of optimal pallidal stimulation sites for dystonic patients in order to evaluate the possible therapeutic implications of connectivity maps obtained from CBP (Neumann et al., 2017; Okromelidze et al., 2020; Reich et al., 2019).

## 2 MATERIALS AND METHODS

### 2.1 Subjects

We employed minimally preprocessed diffusion and structural data of 100 unrelated healthy subjects (males = 46, females = 54, age range 22–36 years) obtained from the HCP repository. Data have been acquired by the Washington University, University of Minnesota and Oxford university (WU‐Minn) HCP consortium. Subject recruitment procedures, informed consent, and sharing of deidentified data were approved by the Washington University in St. Louis Institutional Review Board (IRB) (Van Essen et al., 2013).

### 2.2 Data acquisition

MRI acquisitions were carried out using a custom‐made Siemens 3T “Connectome Skyra” (Siemens, Erlangen, Germany), provided with a Siemens SC72 gradient coil and stronger gradient power supply with maximum gradient amplitude (Gmax) of 100 mT/m (initially 70 and 84 mT/m in the pilot phase), which allows improvement of diffusion‐weighted imaging (DWI).

High‐resolution T1‐weighted MPRAGE images were collected using the subsequent parameters: voxel size = 0.7 mm, TE = 2.14 ms, TR = 2400 ms (Van Essen et al., 2012). DWIs were acquired using a single‐shot 2D spin‐echo multiband Echo Planar Imaging sequence and equally distributed over three shells*(b*‐values 1,000; 2,000; 3,000 mm/s^2^), 90 directions per shell, spatial isotropic resolution of 1.25 mm (Sotiropoulos et al., 2013). More details about experimental setup and preprocessing pipelines are described elsewhere (Glasser et al., 2013; Glasser et al., 2016; Sotiropoulos et al., 2013; van Essen et al., 2012).

### 2.3 Postprocessing

T1‐weighted structural images underwent brain extraction, as well as cortical and subcortical segmentation, implemented by BET, FAST, and FIRST FSL functions, respectively (https://fsl.fmrib.ox.ac.uk/fsl) (Patenaude, Smith, Kennedy, & Jenkinson, 2011; Smith, 2002; Smith et al., 2004), to obtain five‐tissue‐type images which were employed to run MSMT‐CSD. CSD signal modeling estimates white matter fiber orientation distribution function (fODF) from the diffusion‐weighted deconvolution signal using a single fiber response function (RF) as reference (Tournier et al., 2008; Tournier, Calamante, & Connelly, 2007). MSMT‐CSD implement the classical CSD approach by calculating different RFs for gray matter, white matter and cerebrospinal fluid (Jeurissen et al., 2014). RF calculation, fODF estimation, and tractography were implemented using MRtrix3 (www.mrtrix.org).

### 2.4 Whole brain tractography

Whole brain probabilistic tractography was carried out reconstructing 5 million streamlines using default parameters: algorithm = IFOD2 (Tournier, Calamante, & Connelly, 2010), step = 0.5 × voxel size, maximum angle = 90° × step/voxel size, cutoff = 0.05.

### 2.5 Regions of interest

T1‐weighted images were registered to the ICBM 2009b nonlinear asymmetric template employing ANTs symmetric diffeomorphic image registration (SyN) implemented on lead‐DBS v2.3 (Avants, Tustison, & Song, 2009; Horn et al., 2019). We used direct and inverse transformations to register caudate, putamen, nucleus accumbens, GPi, GPe, and STN regions of interest (ROIs) from the CIT168 Reinforcement Learning Atlas to native space of each subject (Pauli, Nili, & Michael Tyszka, 2018).

ROIs of cortical areas and thalamic masks were obtained from the Desikan–Killiany atlas (Desikan et al., 2006) featured in the FreeSurfer software (https://surfer.nmr.mgh.harvard.edu) (Fischl et al., 2002). A modified and improved version of FreeSurfer's *recon‐all* pipeline is part of the minimal preprocessing procedures provided by the HCP repository; further details can be found in Glasser et al. (2013).

**FIGURE 1:**
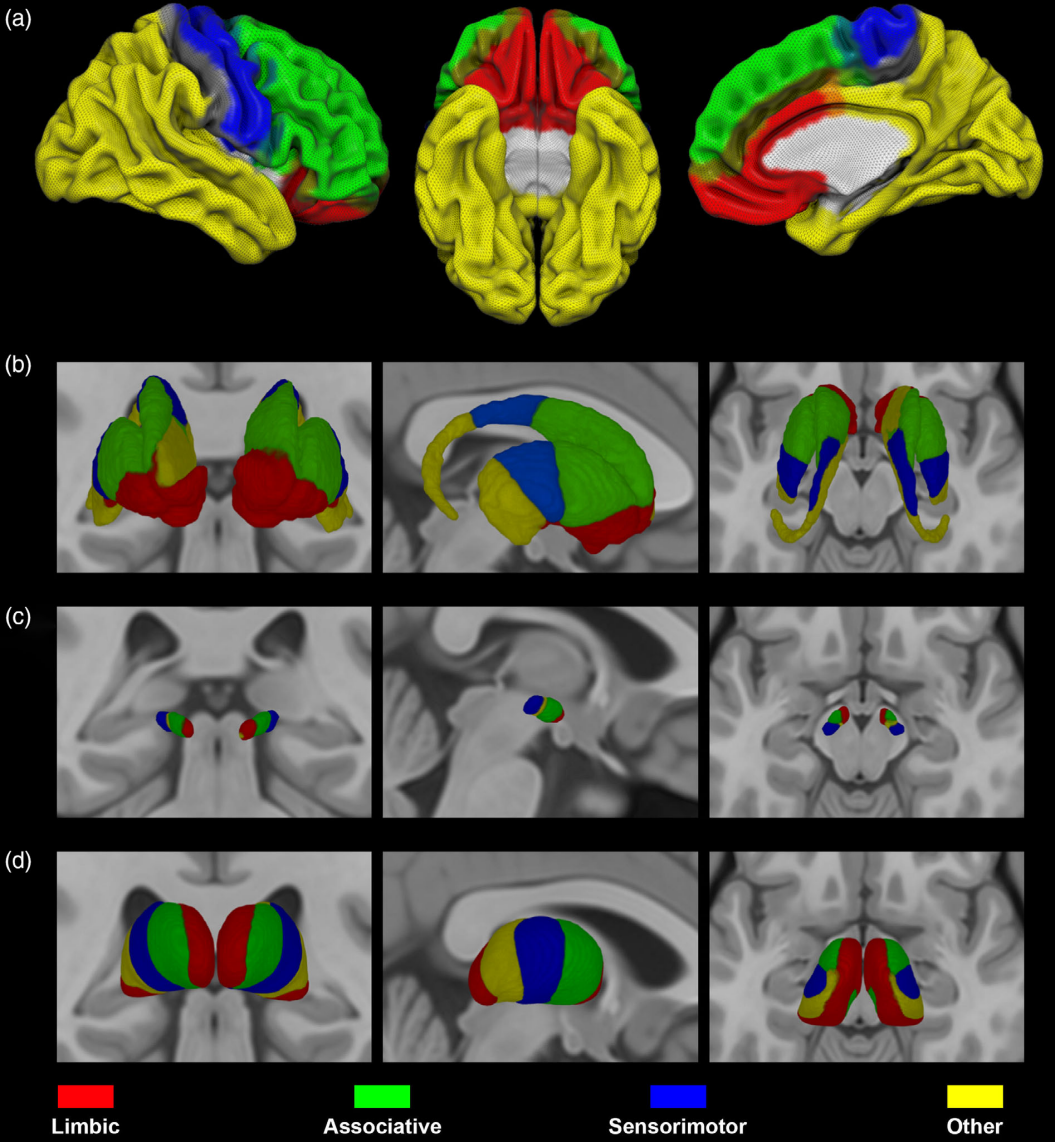
Connectivity‐based parcellation of striatum, subthalamic nucleus (STN), and thalamus. The uppermost row (a) shows 3D lateral and inferior views of the ICBM 2009b nonlinear asymmetric template depicting cortical regions grouped in four functional targets: Limbic (red), associative (green), sensorimotor (blue), other (yellow). In the lower rows, coronal, sagittal, and axial sections of the ICBM 2009b nonlinear asymmetric template showing 3D‐volume rendering of striatal (b), subthalamic (c), and thalamic (d) maximum probability maps. Connectivity maps of striatum, thalamus, and STN follow the color‐code provided for cortical targets. Images are shown in neurological convention

All the obtained masks were then visually inspected and, if needed, manually corrected by a trained neuroanatomist (D. M.). Like in previous studies (Cacciola, Milardi, Bertino, et al., 2019;Patriat et al., 2018; Plantinga et al., 2018), cortical gyral ROIs were merged into four function‐related groups: a limbic group including lateral orbitofrontal cortex, medial orbitofrontal cortex, frontal pole, and anterior cingulate cortex; an associative group, consisting of superior, middle, and inferior frontal gyri; a sensorimotor group, corresponding to precentral gyrus, postcentral gyrus and paracentral lobule; finally, all the remaining cortical ROIs were included in an “other” group. (Figure 1a) (Patriat et al., 2018; Plantinga et al., 2018).

### 2.6 Connectivity‐based parcellation

CBP was carried out for GPi and GPe separately, employing a winner‐takes‐all algorithm (Behrens et al., 2003; Cacciola, Milardi, Basile, et al., 2019; da Silva et al., 2017; Steele et al., 2016). We employed a two‐stage hypothesis‐driven parcellation:

- CBP of the striatum (caudate, putamen and nucleus accumbens), STN and thalamus according to their cortical connectivity using previously mentioned functionally‐related groups as targets, obtaining for each group (limbic, associative, sensorimotor, “other”) striatal, subthalamic, and thalamic connectivity maps.
- CBP of GPi and GPe employing striatal, subthalamic and thalamic connectivity maps obtained in the first stage as targets, thus retrieving distinct parcels for GPi and GPe.

As output, we obtained a total of 12 maps (four maps for three bundles of interest: striatopallidal, subthalamopallidal, pallidothalamic) for GPi and 8 maps for GPe (four maps for two bundles of interest: striatopallidal and subthalamopallidal).

The first parcellation procedure (i) was carried out implementing the following pipeline:

1. Connectivity profiles were obtained by filtering tractograms to obtain tracts of interest.
2. Tracts were mapped to voxels retrieving track density imaging (TDI) maps (Calamante, Tournier, Smith, & Connelly, 2012) which were then multiplied for ROI of GPi and GPe to obtain connectivity density‐weighted maps.
3. TDI maps were normalized by calculating for each map the ratio between its voxels' intensity and its mean intensity value. This was done in order to obtain comparable connectivity density values among different maps.
4. A “winner‐takes‐all” algorithm (*find_the_biggest* FSL command) was applied to normalized maps in order to univocally attribute voxel to targets (Behrens et al., 2003).

All the obtained parcels were registered to the ICBM 2009b nonlinear asymmetric template, binarized and summed up to obtain maximum probability maps (MPMs) reflecting the average parcellation at the whole sample level. Finally, a 50% threshold has been applied to these maps to retain only voxels overlapping in at least half of the sample (da Silva et al., 2017; Domin & Lotze, 2019).

The second analysis (ii) was carried out following the same procedure of (i) with exception of the Step 1. Indeed, we employed seed‐based probabilistic tractography to reconstruct tracts connecting each pallidal segment with connectivity maps obtained from the Step 4 of the first analysis. Given the complex course and configuration of subcortical pathways traversing the GP, we selected specific tracking parameters (1,000 seeds per voxel, step 1.25 × voxel size, angle 30° × step/voxel size, cutoff 0.025). Such parameters were shown to perform well in case of regions characterized by high intravoxel heterogeneity of fiber configuration (Maffei, Sarubbo, & Jovicich, 2019). Finally, volumes and center of gravity (COG) of 50%‐thresholded pallidal MPM have been extracted.

### 2.7 Similarity across different white matter bundles

In order to investigate whether connectivity maps resulting from striatopallidal, subthalamopallidal and pallidothalamic pathways showed similar spatial organization within GP, similarity across homologous parcels (same functional territory) derived from different bundles of interest has been quantified computing the Dice coefficient (Dice, 1945):

$$D=\frac{2|A\cap B|}{\left(|A|+|B|\right)}$$

where *A* and *B* represent the number of voxels of MPMs thresholded at 50%. Specifically, Dice coefficients were computed for each functional cluster (limbic, associative, sensorimotor, “other”), for all the possible pairwise combinations for the GPi (e.g., striatopallidal vs. pallidothalamic, striatopallidal vs. subthalamopallidal, pallidothalamic vs. subthalamopallidal) and GPe (e.g., striatopallidal vs. subthalamopallidal).

### 2.8 Reproducibility across subjects

In order to quantify the interindividual variability of the connectivity maps across subjects, we computed two different similarity measures. In particular, reproducibility of pallidal parcellations was evaluated by calculating the overlap‐by‐label (OBL), which represents a measure of the overlap of each cluster across all datasets, and the total accumulated overlap (TAO) that measures the overall, groupwise overlap for a given parcellation type. Both OBL and TAO are based on the Tanimoto coefficient, which measures the similarity between different sets. Being A and B two images on the same space from different subjects, the Tanimoto coefficient is defined as:

$$T\left(A,B\right)=\frac{N\left(A\cap B\right)}{N\left(A\cup B\right)}$$

where *N* represents the number of voxels (Crum, Camara, & Hill, 2006).

For a group of *m* pairs of images, where *m* represents all the possible pairwise combinations of the same connectivity map *i* across different subjects, OBL is defined as:

$$\mathrm{OBL}=\frac{\sum_{m}\alpha_{i}N\left(A_{i}\cap B_{i}\right)}{\sum_{m}\alpha_{i}N\left(A_{i}\cup B_{i}\right)}$$

In order to avoid overestimation of larger parcels, a weighting coefficient *α* is employed. We defined *α* as the inverse of the mean of the absolute value of volumes for *A* and *B*:

$$\alpha =\frac{2}{\left|A\right|+|B|}$$

On the other hand, for a number *n* of clusters from a given parcellation TAO is defined as:

$$\mathrm{TAO}=\frac{\sum_{m}\sum_{i=1:n}\alpha_{i}N\left(A_{i}\cap B_{i}\right)}{\sum_{m}\sum_{i=1:n}\alpha_{i}N\left(A_{i}\cup B_{i}\right)}$$

(da Silva et al., 2017; Traynor et al., 2010).

For both similarity indices, the result is a number ranging between 0 and 1, where a value closer to 1 indicates higher similarity and a value closer to 0 indicates high dissimilarity.

### 2.9 Quantitative analysis

A quantitative volume‐based measure for each GP parcel at subject level was estimated by calculating the streamline density index (SDI), defined as the percentage ratio between each map volume and the GPi or GPe ROI volume, respectively, as reported in the following formula (Theisen et al., 2017):

$$\mathrm{SDI}=\frac{v}{V_{\mathrm{ROI}}}\times 100$$

where *v* is the volume (in voxels) of parcel obtained after the application of the hard segmentation algorithm and *V*_ROI_ is the volume (in voxels) of the ROI of either GPi or GPe.

In order to assess statistically significant lateralization for each connectivity map, permutation tests based on a *t*‐statistic were performed using the connectivity profiles as defined by SDI of each hemisphere gathered from each subject. Then, 50,000 permutations were used to estimate the distribution of the null hypothesis, alpha level was set to .05, and the “*t*‐max” method was adopted to correct the *p*‐values of each variable for multiple comparisons (Blair & Karkiski, 1993).

Moreover, a lateralization index (LI) has been computed to identify eventual side dominance at subject level (Parker et al., 2005; Wang et al., 2020). LI was calculated for each connectivity map in the native space according to the following formula:

$$\mathrm{LI}=\frac{\text{Left}-\text{Right}}{\text{Left}+\text{Right}}$$

Positive values stand for left‐lateralization (LI > 0.1), while negative values suggest right‐lateralization (LI < −0.1).

### 2.10 Spatial relations between COG and optimal stimulation sites in dystonia

In order to quantify the spatial relations between GPi sensorimotor maps and optimal stimulation sites for dystonic patients provided by previously published studies, the Euclidean distances (in mm) between the COG of the GPi sensorimotor maps and the literature‐based coordinates of the optimal stimulation sites were computed. Specifically, six optimal stimulation sites were considered:

- the peak of theta activity, defined as the centroid of contact pair locations ranging above the 95th percentile in theta amplitude;
- average active electrode position corresponding to the centroid of all active stimulation contacts, both provided by the study of Neumann et al., conducted on 32 patients with cervical dystonia (Neumann et al., 2017);
- optimal stimulation site provided by Schönecker et al., retrospectively, studying electrode locations on 15 patients with cervical dystonia (Schönecker et al., 2015);
- optimal stimulation site proposed by Starr et al. (Starr, 2002) based on 75 GPi implants in dystonic patients;
- coordinates of the stimulation site whose related volume of tissue activated (VTA) gathered the highest correlation with unified dystonia rating scale (UDRS) improvement in a cohort of 39 implanted patients suffering from idiopathic isolated generalized dystonia (Okromelidze et al., 2020);
- coordinates of an anti‐dystonic sweet spot derived from VTA modeling of 105 implanted patients affected by isolated‐generalized, segmental, and cervical dystonia (Reich et al., 2019).

## 3 RESULTS

### 3.1 Corticostriatal, corticosubthalamic, and thalamocortical parcellation

Striatum, STN, and thalamus have been parcellated according to their cortical structural connectivity. A 3D rendering of MPMs with 50% population threshold is depicted in Figure 1. The spatial organization of connectivity maps described for these subcortical structures is in line with previous investigations (Patriat et al., 2018; Plantinga et al., 2018; Tziortzi et al., 2014).

For the striatum, the limbic territory occupies the nucleus accumbens as well as the ventral portion of caudate and putamen. Connectivity to associative areas segregated in the anterior portion of caudate and putamen, posteriorly to their limbic territory; sensorimotor connectivity involves posterior portion of striatum while “other” regions were localized in the caudal tip of putamen and in the tail of caudate nucleus.

The STN parcellation resulted in an anterior limbic region, a dorsal associative region, a ventral “other” region, both located in the intermediate portion of the nucleus, and a posterior sensorimotor region.

For thalamus, connectivity with limbic cortical areas involves the medial half, extending to the most posterior portion, while associative connections segregate in the anterolateral thalamus, followed posteriorly by the sensorimotor and the “other” parcels which were located in a posterior lateral region.

### 3.2 GPi and GPe parcellation

We successfully reconstructed the striatopallidal, subthalamopallidal and pallidothalamic pathways and their related limbic, associative, sensorimotor, and "other" parcels in all subjects (*n* = 100, 100%).

Spatial organization of MPMs within GPi and GPe is described below. Volumes (in mm^3^) and COG (x, y, z) are provided in Table 1, while SDI values (mean ± *SD*) of both GPi and GPe are summarized in Table 2.

**TABLE 1:** The table shows volumes in mm^3^ and COG in MNI space coordinates of GPi and GPe connectivity clusters obtained from striatopallidal, subthalamopallidal and pallidothalamic parcellations. Both volumes and COG have been assessed after applying a 50% threshold on average maps registered to MNI ICBM 2009b nonlinear asymmetric template

	GPi		GPe	
	Volume (mm^3^)	COG (x,y,z)	Volume (mm^3^)	COG (x,y,z)
*Striatopallidal pathway*				
L limbic	320	−14.2, −3.1, −5.6	452	−14.6, 2.3, −2.6
R limbic	172	15.1, −2.4, −4.9	368	15.5, 3.4, −1.5
L associative	234	−15.6, −6.1, −3.9	488	−17.4, −0.5, 2.21
R associative	160	15.7, −4.7, −2.9	420	18.5, 0.5, 2.3
L sensorimotor	167	−19.9, −9.1, −3.4	479	−21.3, −5.9, 1
R sensorimotor	129	20, −8.1, −2.6	430	21.2, −3.4, 0.3
L other	250	−18.6, −6.1, −4.5	400	−21.4, −4.8, −3.1
R other	157	20.2, −6.3, −4.1	369	22, −4.3, −2.3
*Subthalamopallidal pathway*				
L limbic	252	−14, −3.4, −4.9	730	−16.6, 0.2, −1.2
R limbic	219	14.5, −2.5, −4.6	524	16.5, 2.2, −0.7
L associative	254	−17.3, −6.1, −3.6	255	−16.8, −0.8, 2.9
R associative	186	17, −4.6, −3.3	288	17.5, 1.2, 1.8
L sensorimotor	208	−20.3, −8.6, −4.3	696	−20.6, −4.4, −0.1
R sensorimotor	210	20.6, −7.4, −3.9	684	21.2–3.4, 0.3
L other	126	−14.3, −3, −6.4	558	−17.8, −0.6, −1.7
R other	130	19, −6.5, −2.8	378	19.5, −1.2, 1.1
*Pallidothalamic pathway*				
L limbic	255	−13.7, −2.7, −5.6	/	/
R limbic	218	14.9, −2.3, −4.8	/	/
L associative	235	−16.4, −6.2, −3.3	/	/
R associative	163	16.3, −5.2, −3.1	/	/
L sensorimotor	162	−17.9, −8.1, −4.4	/	/
R sensorimotor	151	19.5. ‐7.5, −3.1	/	/
L other	306	−18.7, −6.2, −5	/	/
R other	259	19.8, −6.1, −4.2	/	/

Abbreviations: COG, center of gravity; GP, globus pallidus; GPe, GP external; GPi, GP internal.

**TABLE 2:** The table shows mean and *SD* values (in %) of SDI referred to connectivity clusters of GPi and GPe according to their striatopallidal, subthalamopallidal, and pallidothalamic connectivity

SDI				
	GPi		GPe	
	Mean (%)	*SD* (%)	Mean (%)	*SD* (%)
*Striatopallidal pathway*				
L limbic	**32**.72*	5.74	**26**.83*	3.53
R limbic	**27**.**67**	10.29	**23**.**86**	3.75
L associative	**24**.**70**	3.92	28.61	3.55
R associative	**26**.48*	8.60	28.19	3.67
L sensorimotor	**18**.**65**	3.59	**26**.**11**	2.97
R sensorimotor	**21**.18*	6.83	**27**.54*	2.86
L other	23.92	5.63	**18**.**45**	3.20
R other	24.68	10.15	**20**.34*	3.87
*Subthalamopallidal pathway*				
L limbic	**27**.**65**	8.97	28.76	8.47
R limbic	**30**.65*	7.60	26.10	6.85
L associative	**28**.65*	5.99	15.74	3.43
R associative	**25**.**54**	7.09	16.80	4.94
L sensorimotor	**23**.**30**	5.22	**28**.**63**	7.95
R sensorimotor	**25**.46*	5.78	**32**.23*	8.01
L other	20.39	7.39	**21**.18*	7.57
R other	18.30	5.33	**17**.**13**	4.73
*Pallidothalamic pathway*				
L limbic	**25**.**01**	4.96	/	/
R limbic	**27**.37*	5.05	/	/
L associative	**26**.03*	5.18	/	/
R associative	**23**.**25**	4.12	/	/
L sensorimotor	19.01	3.84	/	/
R sensorimotor	20.39	4.80	/	/
L other	29.86	4.66	/	/
R other	28.99	4.63	/	/

*Note:* Significant left–right differences are indicated by asterisks.

Abbreviations: GP, globus pallidus; GPe, GP external; GPi, GP internal; SDI, streamline density index.

### 3.3 Striatopallidal parcellation

**FIGURE 2:**
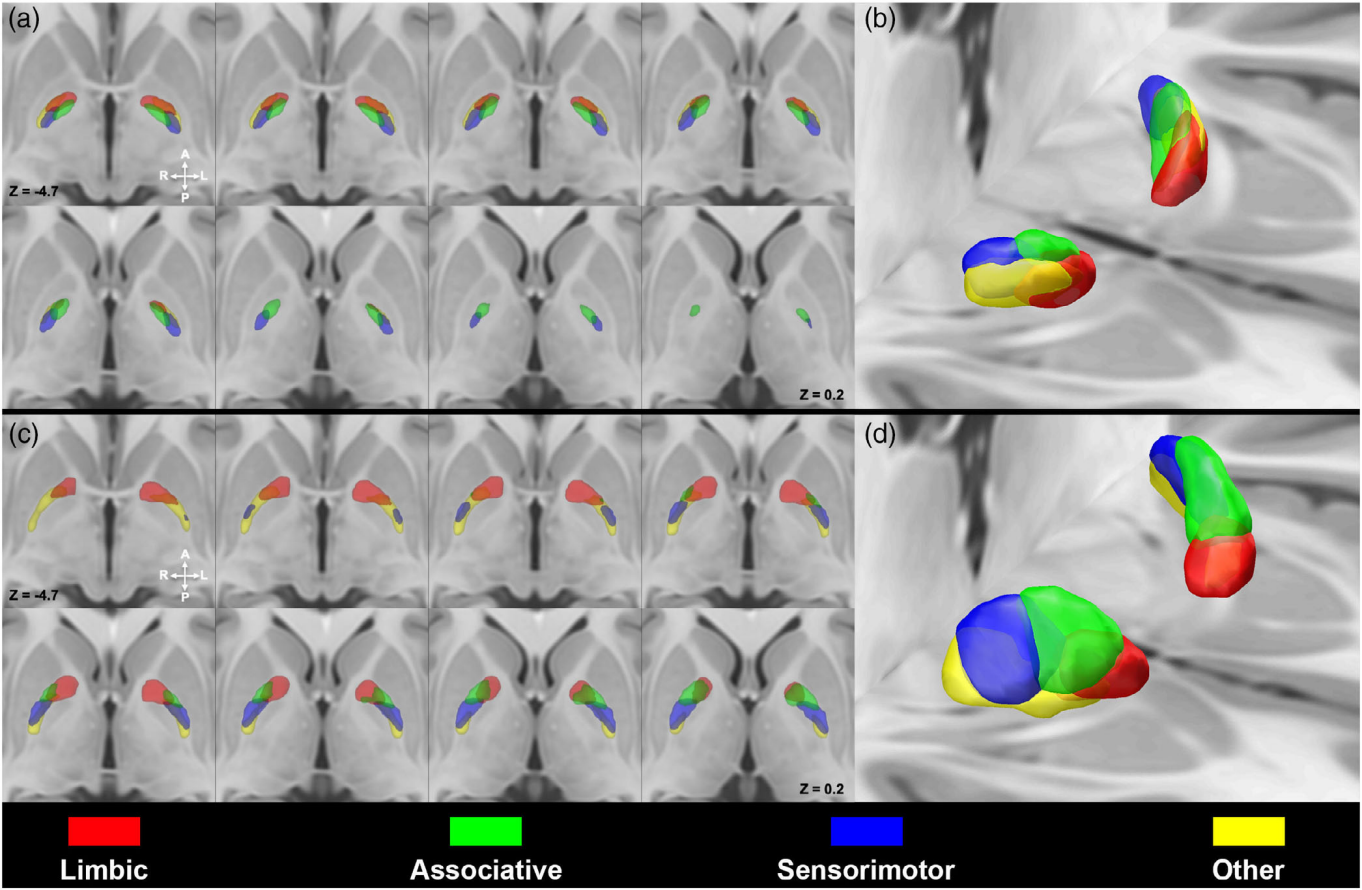
Striatopallidal pathway within the globus pallidus (GP). Multiple axial sections depicting 50% thresholded connectivity maps derived from the parcellation of the internal GP (GPi) (a) and external GP (GPe) (c) according to the striatopallidal pathway, superimposed on the ICBM 2009b nonlinear asymmetric template. 3D volume rendering of GPi (b) and GPe (d) maximum probability maps (MPMs) are provided (images are shown in neurological convention). Connectivity maps were labeled as follows: limbic (red), associative (green), sensorimotor (blue), other (yellow)

Maps obtained from striatopallidal connectivity are depicted in Figure 2. In the GPi (Figure 2a, Supplementary Figure S1a), MPMs were topographically organized according to the following spatial distribution: the limbic territories occupied the anterior region with associative connectivity maps segregating behind, while sensorimotor and “other” regions were located in the posteromedial and posterolateral regions of the nucleus, respectively.

In the GPe (Figure 2c, Supplementary Figure S1b), the limbic territories lain in the most anterior part of the nucleus, the associative regions were located posteriorly, followed by the sensorimotor parcels and the “other” parcels extended from the posterior border of limbic territories to the posterior tip of the GPe.

### 3.4 Subthalamopallidal parcellation

**FIGURE 3:**
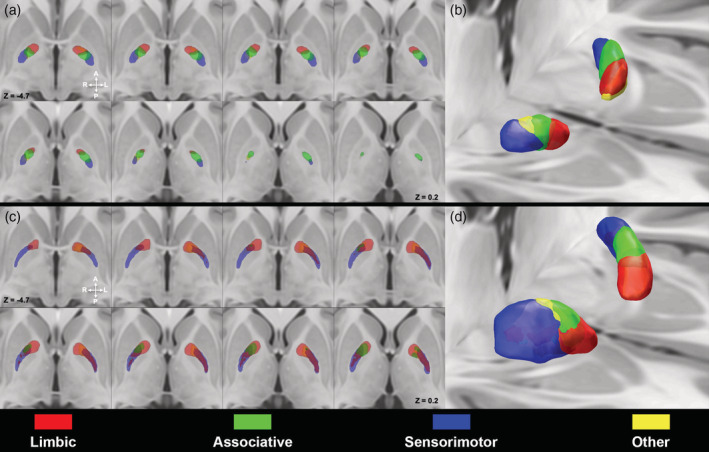
Subthalamopallidal pathway within the globus pallidus (GP). Multiple axial sections of the ICBM 2009b nonlinear asymmetric template depicting 50%‐thresholded probabilistic maps of the internal segment of GP (GPi) (a) and external segment of GP (GPe) (c) obtained according to subthalamopallidal pathway. 3D volume rendering of GPi (b) and GPe (d) maximum probability maps (MPMs) are provided (images are shown in neurological convention). Connectivity maps were labeled as follow: limbic (red), associative (green), sensorimotor (blue), other (yellow)

After mapping, the subthalamopallidal connections in the GPi, limbic, associative, and sensorimotor maps resulted to be arranged along the anteroposterior axis of the nucleus (Figure 3a, Supplementary Figure S2a). The “other” parcels were located anteriorly on the left, while on the right they were placed between associative and sensorimotor clusters.

In GPe, the limbic territories were located in the most anterior aspect with the associative territories segregating behind, followed by the sensorimotor territories, which extended from the posterior boundary of limbic territories to the caudal end of the nuclei. Finally, the “other” maps occupied the intermediate aspect of the nucleus (Figure 3c, Supplementary Figure S2b).

### 3.5 Pallidothalamic parcellation

**FIGURE 4:**
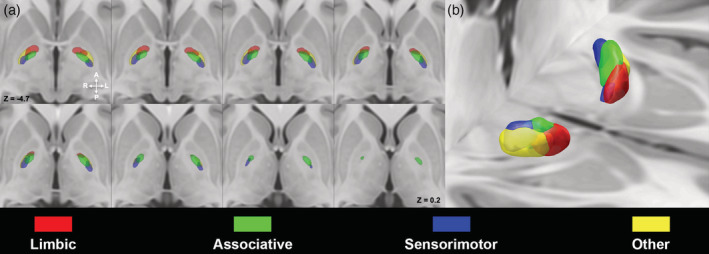
Pallidothalamic pathway within the globus pallidus (GP). Multiple axial slices (a) and 3D volume rendering (b) depicting 50% thresholded connectivity maps of the internal GP (GPi) derived from the pallidothalamic tract on the ICBM 2009b nonlinear asymmetric template. Image in (b) is shown in neurological convention. Color‐coded as follows: limbic (red), associative (green), sensorimotor (blue), other (yellow)

Spatial organization of maps derived from pallidothalamic connectivity was similar to those reported for striatopallidal parcellation (Figure 4a). Limbic regions were located in the most anterior part of GPi, associative territories segregated posteriorly in the medial intermediate region, together with “other” parcels which resulted to be located in the lateral intermediate aspect; the sensorimotor territories occupied the most posterior region of the nucleus (Supplementary Figure S3a).

**FIGURE 5:**
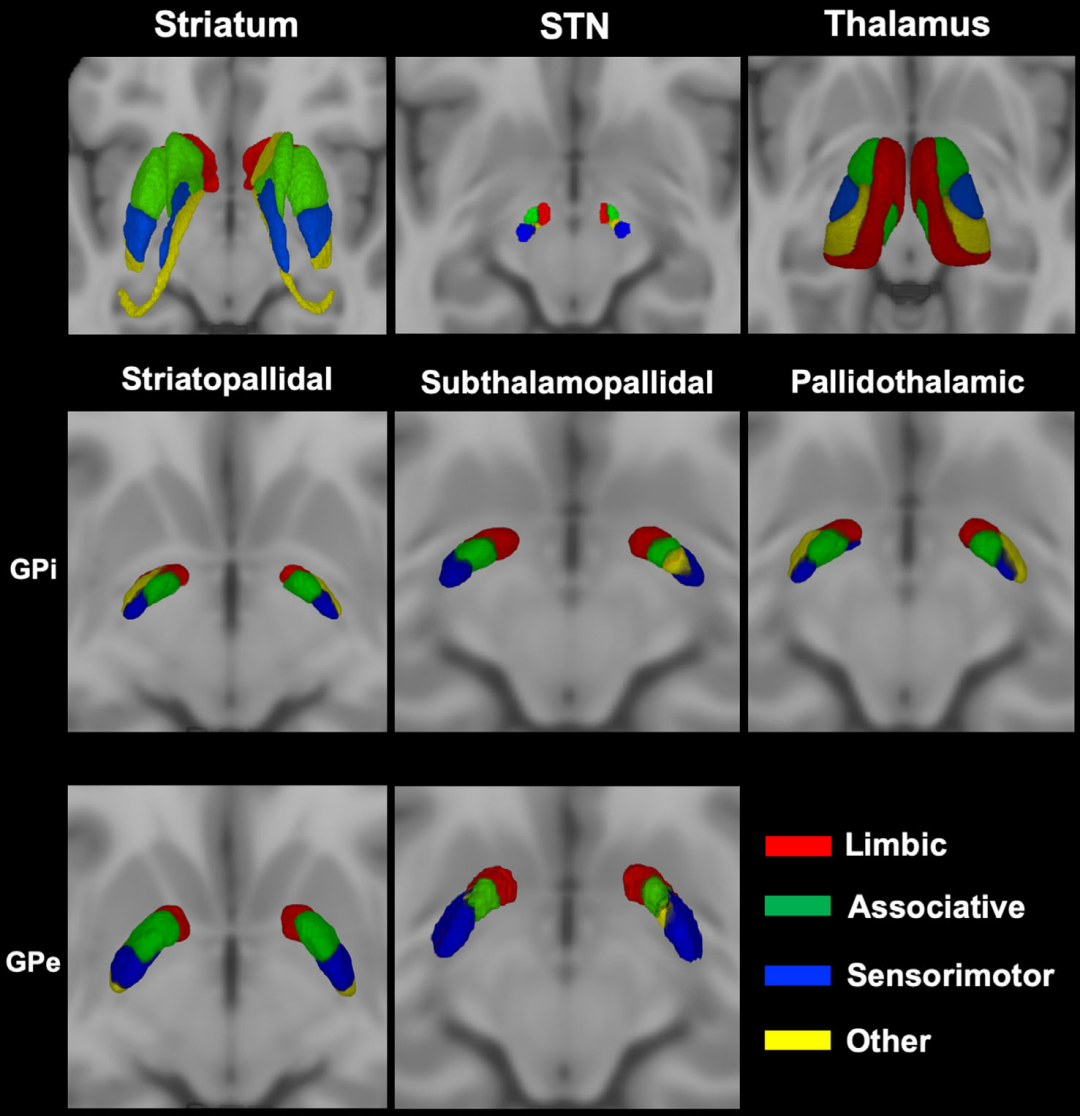
3D volume rendering of 50% thresholded connectivity maps of internal GP (GPi) and external GP (GPe) according to striatopallidal, pallidothalamic, and subthalamopallidal pathways. The uppermost row shows maximum probability maps (MPMs) derived from connectivity‐based parcellation (CBP) of the striatum, subthalamic nucleus (STN) and thalamus according to their cortical connectivity profiles. Connectivity maps of functional territories of these nuclei have been then used to perform CBP of GPi and GPe according to their connectivity patterns (striatopallidal, subthalamopallidal, and pallidothalamic pathways) as shown in the lower rows. Images are shown in neurological convention

3D‐volume rendering of GPi and GPe MPMs derived from different bundles of interest are depicted in Figure 5.

### 3.6 Similarity across parcellation schemes

Dice coefficient values referred to functionally homologous connectivity maps on the standard space are summarized in Table 3.

**TABLE 3:** The table shows Dice coefficients accounting for similarity between couples of functionally homologous clusters of GPi and GPe obtained from different pathways of interest. Dice coefficients have been computed according to the following combinations: pallidothalamic/subthalamopallidal, striatopallidal/subthalamopallidal, and pallidothalamic/striatopallidal for GPi. Dice coefficients between striatopallidal and subthalamopallidal clusters have been assessed for GPe

Dice coefficient				
	GPi			GPe
	Thalamus/STN	Striatum/STN	Thalamus/striatum	Striatum/STN
L limbic	0.81	0.75	0.91	0.67
R limbic	0.86	0.76	0.84	0.78
L associative	0.78	0.65	0.80	0.65
R associative	0.71	0.63	0.80	0.71
L sensorimotor	0.60	0.73	0.67	0.66
R sensorimotor	0.61	0.58	0.80	0.68
L other	0.24	0.26	0.81	0.37
R other	0.47	0.45	0.74	0.28

Abbreviations: GP, globus pallidus; GPe, GP external; GPi, GP internal; STN, subthalamic nucleus.

Regarding the GPi, Dice coefficients have been computed for the following combinations: pallidothalamic and subthalamopallidal maps (limbic: Left = 0.81, Right = 0.86; associative: Left = 0.78, Right = 0.71; sensorimotor: Left = 0.60, Right = 0.61; “other”: Left = 0.27, Right = 0.47), striatopallidal and subthalamopallidal maps (limbic: Left = 0.75, Right = 0.76; associative: Left = 0.65, Right = 0.63; sensorimotor: Left = 0.73, Right = 0.58; “other”: Left = 0.26, Right = 0.45) and striatopallidal and pallidothalamic maps (limbic: Left = 0.67, Right = 0.78; associative: Left = 0.80, Right = 0.80; sensorimotor: Left = 0.67, Right = 0.80; “other”: Left = 0.81, Right = 0.74). As regard the GPe, Dice coefficients have been assessed between maps obtained from striatopallidal and subthalamopallidal parcels (sensorimotor: Left = 0.66, Right = 0.68; associative: Left = 0.65, Right = 0.71; limbic: Left = 0.67, Right = 0.78; “other”: Left = 0.37, Right = 0.28).

### 3.7 Reproducibility analysis

We calculated two different measures of similarity (OBL and TAO) to evaluate reproducibility of CBP for each pathway of interest (Table 4). TAO values were comparable across different pathways of interest both for GPi (striatopallidal: Left = 0.39, Right = 0.37; subthalamopallidal: Left = 0.34, Right = 0.37; pallidothalamic: Left = 0.36, Right = 0.35) and GPe (striatopallidal: Left = 0.46, Right = 0.41; subthalamopallidal: Left = 0.34; Right = 0.33) reaching higher values in the latter. OBL values were similar across different pathways of interest being overall higher for associative and limbic clusters both in GPi (striatopallidal, associative: Left = 0.42, Right 0.39; limbic: Left = 0.46, Right = 0.40; subthalamopallidal, associative: Left = 0.41, Right 0.34; limbic: Left 0.46, Right 0.39; pallidothalamic, associative: Left = 0.39, Right = 0.34; limbic: Left = 0.40, Right = 0.44) and in GPe for striatopallidal (associative: Left = 0.50, Right 0.47; limbic = 0.50, Right = 0.40) pathway. OBL values were higher for limbic clusters across pathways of interest and the highest values were found for GPe connectivity maps.

**TABLE 4:** The table shows values of intersubject similarity measures of GPi and GPe connectivity maps. OBL values of limbic, associative, sensorimotor, and “other” territories measuring cluster‐wise reproducibility are reported in the upper part of the table. TAO values, which measure parcellation‐wise reproducibility, are reported in the lower most row for each pathway of interest

	GPi						GPe			
	Striatopallidal		Subthalamopallidal		Pallidothalamic		Striatopallidal		Subthalamopallidal	
OBL	Left	Right	Left	Right	Left	Right	Left	Right	Left	Right
Limbic	0.46	0.40	0.39	0.39	0.40	0.41	0.50	0.40	0.41	0.38
Associative	0.42	0.39	0.41	0.34	0.39	0.34	0.50	0.47	0.29	0.27
Sensorimotor	0.34	0.35	0.35	0.38	0.27	0.29	0.47	0.46	0.39	0.42
Other	0.35	0.36	0.21	0.25	0.39	0.39	0.36	0.34	0.29	0.26
TAO	0.39	0.37	0.34	0.34	0.36	0.35	0.46	0.41	0.34	0.33

Abbreviations: GP, globus pallidus; GPe, GP external; GPi, GP internal; OBL, overlap‐by‐label; TAO, total accumulated overlap.

### 3.8 Hemispheric asymmetries

Interhemispheric (left vs. right) asymmetries (Table 2) were found for the connectivity profiles, expressed as SDI, between the following connectivity maps: limbic clusters showed significant left‐lateralization for striatopallidal both in GPi (*p* < .001) and GPe (*p* < .001) while being right lateralized in subthalamopallidal (*p* < .05) and pallidothalamic pathways in GPi (*p* < .05). Associative clusters were left‐lateralized for pallidothalamic (*p* < .001) and subthalamopallidal (*p* < .05) and right‐lateralized for striatopallidal pathways in GPi (*p* < .05); no significant lateralization was found for the GPe. A significant right‐lateralization was observed for sensorimotor clusters derived from striatopallidal and subthalamopallidal pathways both in GPi (*p* < .001 and *p* < .05) and in GPe (*p* < .05 and *p* < .05) but not for the pallidothalamic pathway. “Other” clusters were not significantly lateralized in GPi while being right lateralized and left lateralized in striatopallidal (*p* < .05) and subthalamopallidal (*p* < .001) pathways respectively in GPe.

**FIGURE 6:**
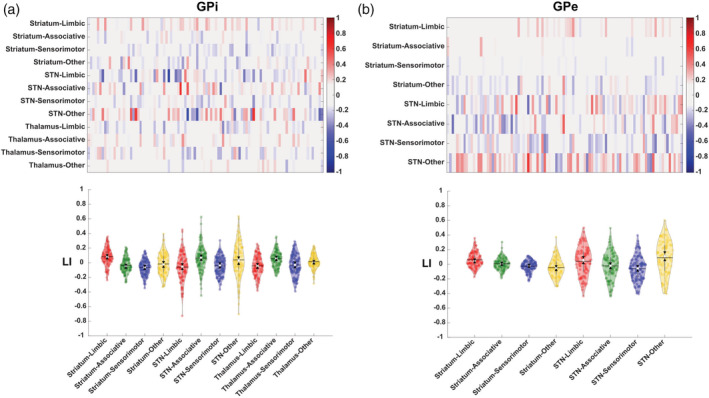
Distribution of lateralization index (LI) computed according to streamlines density index values at subject level. (a) The matrix reports LI values for each connectivity patterns and for each subject. Left‐lateralized maps (LI > 0.1) are depicted in red while right‐lateralized maps (LI < −0.1) are portrayed in blue. The color bar refers to the strength of lateralization: the higher the color intensity, the higher is the degree of hemispheric dominance. (b) Violin plots report the distribution of the LI and the degree of lateralization. The violins ad data points are colored according to the following color coding: associative (green), limbic (red), sensorimotor (blue), other (yellow). The solid black lines of the violins depict the mean value of the distribution; the white circle depicts the median point and the black triangles represent the notch as the 95% confidence interval around the median. The vertical gray line depicts the 25th and 75th percentiles

In addition, a LI for each subject was calculated on SDI of probabilistic maps obtained from different bundles of interest, in order to investigate the distribution of left‐ or right‐sided dominance on the whole sample (Figure 6).

GPi striatopallidal parcellation reported left‐dominance of limbic SDI in the 42% of the sample while being right‐dominant in 10%. Left‐lateralized associative clusters were observed in the 11% of the whole sample; on the other hand, the 22% resulted to be right‐lateralized. Sensorimotor SDI values were left dominant on the 6% and right dominant in the 42% of subjects. Finally, “other” clusters resulted to be left‐lateralized in the 22% and right‐lateralized in the 29% of the whole sample.

LI calculated for clusters obtained from GPi subthalamopallidal parcellation showed limbic left‐dominance in the 21% of the sample and right‐dominance in the 36%. Associative SDI showed left lateralization in the 39% of subjects and right lateralization in the 15%. A sensorimotor left‐lateralization has been reported in the 11% of the cases while a right‐lateralization was noted in the 38%. SDI values referred to “other” clusters were left‐lateralized in the 38% and right‐lateralized in the 25% of the whole group.

Among maps obtained from pallidothalamic connectivity, limbic clusters were left‐lateralized in the 17% of the subjects while being right‐lateralized in the 35%. Left‐dominance was a feature of the 33% of subjects and right dominance of the 10% for associative SDI values. Sensorimotor SDI was left‐lateralized in the 20% of cases while right‐lateralized in the 31%. Analysis of asymmetry among “other” SDI values resulted in left‐lateralization in the 19% of subjects and in right‐lateralization in the 11%.

GPe striatopallidal parcellation provided limbic left‐lateralization in the 32% of subjects while right‐lateralization was reported in the 3%. Associative SDI values showed left‐dominance in the 12% of cases and right dominance in the 7%. SDI values associated with sensorimotor clusters resulted to be left‐lateralized in the 4% of the subjects and right‐lateralized in the 17%. Evaluation of asymmetry for SDI values of “other” clusters revealed left‐dominance in the 14% and right‐dominance in the 31% of the whole sample.

Clusters retrieved from subthalamopallidal parcellation of the GPe demonstrated limbic left‐lateralization in the 41% while right lateralization was observed in the 24% of the total subjects. Associative left‐dominance was noticed in the 23%; on the other hand, 31% of the sample exhibited rightdominance. Sensorimotor SDI was left‐lateralized in the 17% of cases and right‐lateralized in the 41%. Finally, “other” clusters were found to be leftdominant in 52% of the subjects and rightdominant in the 23%.

### 3.9 Spatial relations between sensorimotor maps and pallidal optimal stimulation sites in dystonia

**FIGURE 7:**
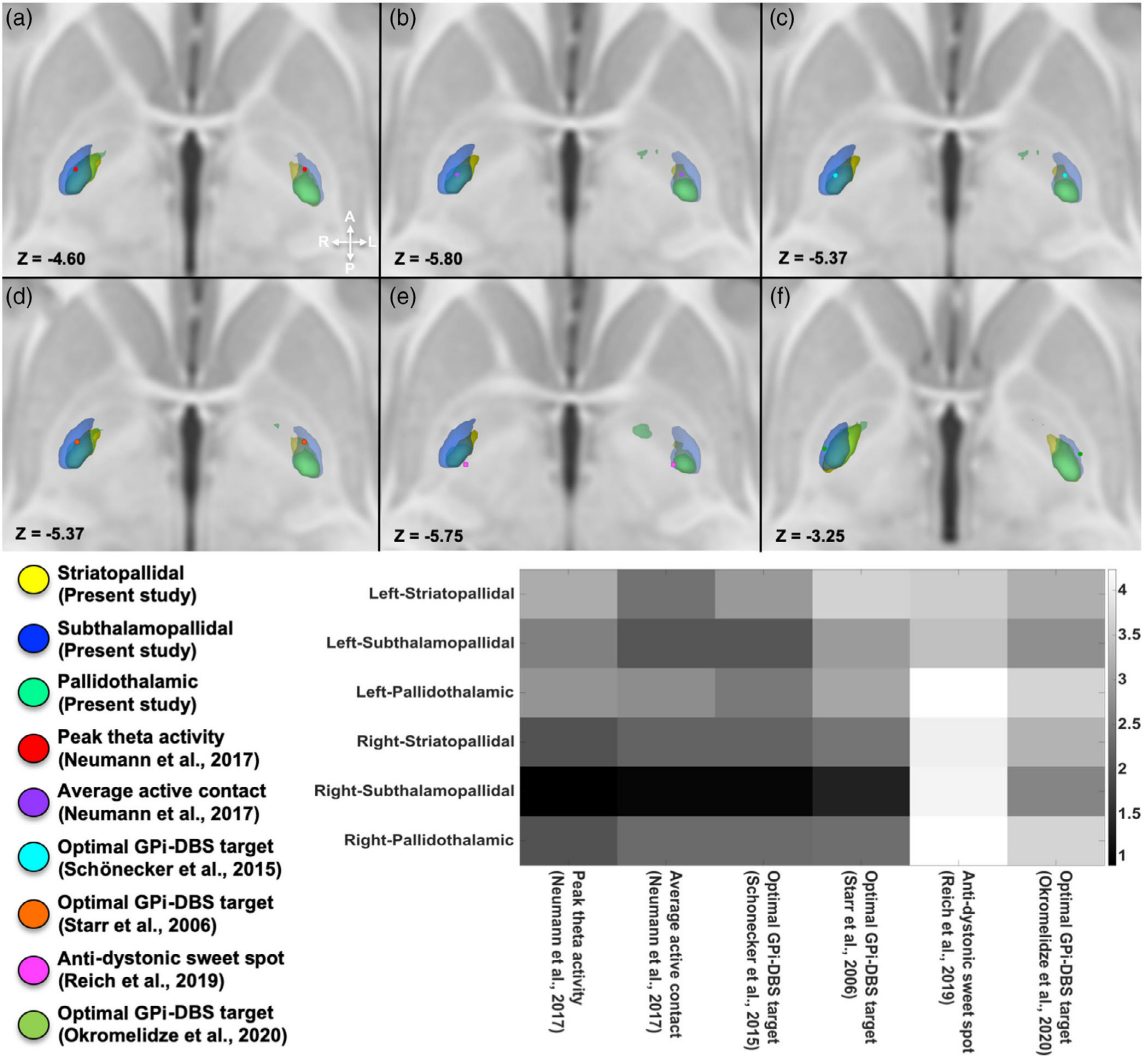
Spatial relationship between sensorimotor connectivity maps, site of peak theta activity and pallidal optimal stimulation site for dystonia, as found in Neumann et al. (2017), Okromelidze et al. (2020), Reich et al. (2019), Schönecker et al. (2015), and Starr (2002), superimposed on the ICBM 2009b nonlinear asymmetric template. In (a), the spatial relation between the site of peak theta activity (red circle) and striatopallidal (yellow), subthalamopallidal (blue), and pallidothalamic (green) maps is shown. In (b), the spatial relation between average optimal deep brain stimulation (DBS) electrode position (violet circle) and subthalamopallidal (blue), pallidothalamic (green) and striatopallidal (yellow) maps is depicted. In (c) and (d), the spatial relationships between the sensorimotor connectivity maps and pallidal optimal stimulation sites for dystonia identified respectively by Schöneker et al. (c) (cyan circle) and Starr et al (d) (orange circle) superimposed on the ICBM 2009b nonlinear asymmetric template are shown. Finally, coordinates of pallidal sweet spot provided by Reich et al. (purple circle) and coordinates associated with best clinical outcomes in Okromelidze et al. (green circle) are depicted in (e) and (f), respectively. The matrix located at the bottom right of the figure shows the Euclidean distances between the center of gravity (COGs) of the striatopallidal, subthalamopallidal, and pallidothalamic sensorimotor maps and the coordinates of all the optimal stimulation sites shown in 2D axial sections

Spatial relations between the COGs of sensorimotor maps and those of different DBS optimal stimulation sites have been assessed by computing the Euclidean distances between them. Peak of theta activity and the average active lead position resulted to be located within striatopallidal, subthalamopallidal and pallidothalamic maps; such stimulation points resulted to be the closest to COG of MPM, in particular to those derived from the subthalamopallidal parcellation (peak theta: Left = 3.11 mm, Right = 0.92 mm; active lead location: Left = 2.10 mm, Right =1.01 mm), followed by pallidothalamic (peak theta: Left = 2.74 mm, Right = 1.95 mm; active lead location: Left = 1.97 mm, Right = 2.04 mm) and striatopallidal (peak theta: Left = 3.11, Right = 1.96; active lead location: Left = 2.83 mm, Right = 2.11 mm) connectivity maps (Figure 7).

In addition, we also considered other four optimal stimulation sites as provided in (Okromelidze et al., 2020; Reich et al., 2019; Schönecker et al., 2015; Starr, 2002). Similar results have been obtained for coordinates derived from (Starr, 2002) and (Schönecker et al., 2015) and derived sensorimotor MPMs (Figure 7) with subthalamopallidal COG (Schönecker: Left = 2.67 mm, Right = 0.86 mm; Starr: Left = 3.03 mm; Right = 1.83 mm) being the closest, followed by pallidothalamic (Schönecker: Left = 1.66 mm, Right = 1.57 mm; Starr: Left = 3.82 mm, Right: 3.13 mm) and striatopallidal maps (Schönecker: Left = 3.23 mm, Right = 1.62 mm; Starr: Left = 3.81 mm, Right = 3.07 mm) both showing very similar distances. Finally, distances between COG of sensorimotor connectivity maps and coordinates provided by (Reich et al., 2019) and (Okromelidze et al., 2020) resulted to be greater for striatopallidal (Reich: Left = 3.56, Right = 4.02; Okromelidze: Left = 3.20, Right = 3.25), subthalamopallidal (Reich: Left = 3.41, Right = 4.09; Okromelidze: Left = 2.78, Right = 2.65) and pallidothalamic (Reich: Left = 2.67, Right = 4.23; Okromelidze: Left = 5.18, Right = 3.67) pathways, since such stimulation points were located at the posteromedial and poster‐lateral borders of GPi, respectively.

## 4 DISCUSSION

One of the main aims of the present study was to investigate the topographical arrangement of striatopallidal, subthalamopallidal, and pallidothalamic pathways within the GPi and GPe, by using tractography‐based CBP on 100 unrelated healthy subjects.

The most accepted model on basal ganglia functional anatomy, mainly derived from multimodal evidence collected from nonhuman primates, suggests high spatial coherence of the cortico‐basal ganglia loops. In other words, this means that connections from functionally homologous regions of the cerebral cortex, striatum, and STN are likely to converge on overlapping, yet identifiable pallidal regions (Alexander et al., 1990; Saga et al., 2017). Although other works investigated the topographical organization of GP, most of them are focused only on a specific pathway and are based on a small number of subjects. In the present work, in order to overcome such limitations and to provide a wider framework, we test the hypothesis that the striatopallidal, subthalamopallidal, and pallidothalamic pathways are spatially coherent and topographically organized within the GP in a large sample of healthy subjects (*n* = 100).

Our results suggest that the general topographical organization of connectivity parcels is highly consistent across the GPi and GPe, regardless of the bundle of interest, corroborating the hypothesis that different pathways, running parallel and in series to one other, may share the same spatial organization pattern (Figure 5). Indeed, we found high similarity among functionally homologous connectivity maps derived from the striatopallidal, subthalamopallidal and pallidothalamic tracts (Table 3).

In other words, we show neuroimaging evidences suggesting the existence of partially segregated yet integrated functional territories within the GPi and GPe, derived from connectivity patterns that maintain a consistent topographical arrangement throughout the entire cortico‐basal ganglia‐thalamo‐cortical circuitry (Figures 1 and 5).

Such findings are in line with tract‐tracing and virus‐tracing studies, which revealed that different cortical areas, projecting to GP via distinct portions of the striatum and STN, delineate a tripartite organization which is similar, albeit not identical between the GPi and GPe (Saga et al., 2017). Indeed, limbic, associative and sensorimotor cortical areas project respectively to anteroventral, anterodorsal and posterior portions of both GPi and GPe (Haber, Groenewegen, Grove, & Nauta, 1985; Hedreen & Delong, 1991; Hoover & Strick, 1993; Joel & Weiner, 1997; Kelly & Strick, 2004; Middleton, 2002; Nauta & Cole, 1978; Shink, Bevan, Bolam, & Smith, 1996; Spooren, Lynd‐Balta, Mitchell, & Haber, 1996). However, despite such a consistent spatial organization, Saga et al. demonstrated that patterns of neuronal labeling in GPe were broader and boundaries of functional territories less‐defined than in GPi, thus proposing that the connectivity patterns within the GPe may be less segregated (Saga et al., 2017). This is in line with our results, which show that, although connectivity parcels of GPi and GPe grossly share a very similar spatial organization, a higher degree of overlap among MPMs of different functional territories could be noticed in the GPe (Figures 2, 3, 4). However, it should be kept in mind that MPMs represent voxels which are most coherently part of each connectivity parcel within our sample, rather than biologically grounded representations of connectivity territories within GP. On the other hand, Dice coefficients show generally lower values in the GPe if compared to GPi, in particular for limbic and associative territories (Table 3), likely suggesting a less marked pattern of segregation in the GPe.

Although several studies suggested such a tripartite organization of the GPi and GPe according to their afferent connections, such pattern of spatial coherence might be no longer maintained in the efferent pallidothalamic tract, likely reaching distinct thalamic nuclei instead of being spatially organized along its anterior–posterior axis (Alexander et al., 1990).

In order to verify the possible spatial, topographical organization of the pallidothalamic tract, similarly to previous studies (Behrens et al., 2003; Middlebrooks, Tuna, Almeida, et al., 2018; Patriat et al., 2018), we parcellated the thalamus according to its cortical connectivity retrieving “functional maps” that are likely to oversimplify the anatomical and functional complexity of thalamic nuclei (Morel, Magnin, & Jeanmonod, 1997). Then, we performed the parcellation of the GPi using the abovementioned thalamic parcellations as tractography targets, founding high spatial consistency between MPMs obtained from the pallidothalamic tract and those obtained from the striatopallidal and subthalamopallidal pathways, suggesting that functional territories within the GPi are likely to be shared by afferent and efferent tracts.

It is worth to note that we could not distinguish ansa lenticularis from lenticular fasciculus which is still beyond the possibilities of conventional diffusion MRI (Alho et al., 2020). Moreover, connections between the GPe and thalamus have not been taken into account, as GPe main afferent and efferent connections involve the striatum and STN (Kita, 2007), despite sparse GPe projections to the ventral lateral nucleus, parafascicular complex, and reticular nucleus have been also described (DeVito & Anderson, 1982; Gittis et al., 2014).

To add further information on the topographical organization of pallidal connectivity parcels we evaluated intersubject reproducibility of CBP for each pathway of interest (Table 4).

In particular, in our reproducibility analysis of pallidal parcellations, we evaluated two overlap metrics that slightly differ from each other in their meaning and interpretation: OBL evaluates the similarity of each cluster obtained with the same procedure across the entire dataset, whereas TAO may be interpreted as a global estimate of the overall, groupwise overlap for a given parcellation type. OBL showed uniform values among different pathways; specifically, limbic and associative reached highest values both for GPi and GPe. It could be interesting to note that, in GPi, subthalamopallidal pathway obtained the highest OBL values for sensorimotor maps, followed by striatopallidal and pallidothalamic pathways suggesting higher reproducibility for the former parcellation. Similarly, TAO values were comparable across different pathways of interest both for GPi and GPe reaching slightly higher values in GPe, thus suggesting that every parcellation type shows similar reproducibility across pathways. In particular, slightly higher values were found for striatopallidal parcellation, followed by pallidothalamic parcellation and subthalamopallidal parcellation. This would suggest that tractography results would lead to higher reproducibility for larger target structures such as striatum and thalamus.

In the present study, we found also evidences of lateralization between different functional territories within the GP as shown by significantly different SDI values which are coherent with subject‐level LI values. In particular, limbic clusters showed significant left‐lateralization for striatopallidal both in GPi and GPe while being right lateralized in subthalamopallidal and pallidothalamic pathways in GPi. Associative clusters were left‐lateralized for pallidothalamic and subthalamopallidal pathways and right‐lateralized for striatopallidal pathway in GPi, no significant lateralization was noticeable in GPe. A significant right‐lateralization was observed for sensorimotor clusters both in GPi and in GPe except for pallidothalamic pathway. “Other” clusters were not significantly lateralized in GPi while being right lateralized and left lateralized in striatopallidal and subthalamopallidal pathways respectively in GPe.

While lateralization of functions of the cerebral cortex has been widely studied (Gotts et al., 2013), functional lateralization of basal ganglia both in term of volume and connectivity has been poorly investigated (Greene et al., 2014; Griffanti et al., 2018; Lenglet et al., 2012). Although the significance of such asymmetries is currently unknown, these findings bear further studies, as these subcortical pathways play a pivotal role in both physiological and pathological conditions.

The results of the present study extend previous findings based on diffusion imaging of the human brain, that are mainly focused on the parcellation of GPi, due to its well‐known pathophysiological implications in movement disorders (Patriat et al., 2018). In particular, a very similar topographical organization was formerly described on 30 healthy subjects in the seminal work by Draganski et al. (2008), despite it was not possible to discern between the GPi and GPe. A similar topographical arrangement has been described for the GPi employing a k‐means (data‐driven) clustering approach, which identified an anterior territory mostly connected to prefrontal cortex and putamen, an intermediate portion mainly connected to GPe and brainstem and a posterior portion connected to the thalamus (da Silva et al., 2017). Such particular spatial organization may be explained by the fact that the structural connectivity to subcortical structures was retrieved as a whole, without distinction between different functional territories. In a recent study, CBP of GPi according to its thalamic connections has been performed on 32 subjects employing a winner‐takes‐all approach, finding a very similar functional topography to that reported in the present study (Ewert et al., 2018).

Finally, Patriat et al. performed functional segmentation of the GPi according to its afferent structural connectivity patterns on 17 patients affected by different movement disorders and one healthy control subject, reporting Dice coefficient values, computed across homologous parcels (same functional territory) derived from different bundles of interest, similar to, even if slightly lower than, those of the present work (Table 3) (Patriat et al., 2018).

More recently, Cacciola et al. investigated the topographical arrangement of the cortico‐pallidal pathway within the GPi and the GPe, showing remarkable differences between the connectivity patterns of these structures. If, on the one hand, the cortico‐GPe connections were more represented than the cortico‐GPi ones, on the other hand, sensorimotor, limbic, and associative connectivity patterns were topographically organized in a similar fashion within the GPi and GPe (Cacciola, Milardi, Bertino, et al., 2019). In the present work, we aimed at investigating a complementary framework characterizing the spatial, topographical arrangement of subcortical connectivity patterns within the GPi and GPe, by combining MSMT‐CSD tractography with a two‐stage hypothesis‐driven CBP approach. Hence, the present work widens the previous scenario providing a comprehensive view on the spatial topographical arrangement of the striatopallidal, subthalamopallidal, and pallidothalamic pathways.

Although all the above‐mentioned findings come from in vivo neuroimaging studies investigating connectivity at the macroscale level, a possible microstructural correlate of such a topographical organization has been provided by anatomical studies using calbindin staining. Indeed, the gradient of histochemical calbindin labeling reveals a strongly labeled anterior region, corresponding to the anterior pole of the GPi and ventral portion of the GPe, a poorly labeled central part of the pallidum and an intermediate labeled territory between the poorly and the strongly labeled region (Karachi et al., 2002). This pattern of calbindin expression has been reported also for the striatum (Karachi et al., 2002) and substantia nigra (Damier, Hirsch, Agid, & Graybiel, 2002), suggesting that these areas may correspond to putative limbic, associative, and sensorimotor functional territories described in primates (Haber, 2016).

As a final remark, in line with several anatomical studies reporting the involvement of functionally heterogeneous parietal, temporal, and occipital cortices in the basal ganglia loops (Middleton & Strick, 1996; Yeterian & Pandya, 1993, 1995), Patriat et al. introduced a “other” region including such cortical areas in addition to the classical tripartite model. Following the same parcellation scheme, we found that this additional “other” parcel has a highly variable spatial organization both in the GPi and GPe and across different bundles of interest. This is reflected by highly variable Dice coefficient values, ranging from 0.27 to 0.81, indicating the highest spatial heterogeneity across different parcellations. This finding might be explained by the wide functional diversity of cortical areas included in the “other” target, likely suggesting that connections with parietal, occipital, and temporal areas are not organized in segregated anatomical territories but may be rather involved in different functional basal ganglia loops. Despite heterogeneity among “other” territories derived from different pathways is remarkable, reproducibility as measured by OBL underlines that intersubject variability of “other” clusters among each pathway of interest is comparable to the rest of functional territories. This suggests that the same spatial configuration is likely to be maintained across subjects in the same pathway of interest.

### 4.1 Spatial relationship between sensorimotor pallidal maps and DBS in dystonia

The results of the present study may have possible therapeutic implications in movement disorders. Approaches similar to that described herein have been employed on patients cohorts for the evaluation of electrodes position and volume of tissue activated (VTA) modeling in respect to putative sensorimotor territory of GPi (Middlebrooks, Tuna, Grewal, et al., 2018; Patriat et al., 2018). Indeed, Middlebrooks et al. conducted a pilot study on 11 PD patients, proposing tractography‐based identification of the sensorimotor territory of the GPi as an independent predictor of DBS outcomes (Middlebrooks, Tuna, Grewal, et al., 2018).

Recent studies have also evaluated the clinical or pathophysiological correlation between DBS electrode location, the associated VTA and related clinical outcomes in dystonia identifying optimal stimulations sites (Neumann et al., 2017; Okromelidze et al., 2020; Reich et al., 2019; Schönecker et al., 2015). Gathering together the findings of these studies, the optimal stimulation site is located within the posteroventral third of the GPi, likely corresponding to its sensorimotor functional territory (Neumann et al., 2017; Okromelidze et al., 2020; Reich et al., 2019). In order to investigate the possible clinical or pathophysiological relevance of our results, we evaluated the spatial relationship between sensorimotor connectivity maps derived respectively from the striatopallidal, subthalamopallidal, and pallidothalamic pathways and optimal stimulation sites in the GPi as previously identified in dystonic patients, obtaining similar, spatially coherent results as shown in Figure 7 (Neumann et al., 2017; Okromelidze et al., 2020; Reich et al., 2019; Schönecker et al., 2015; Starr et al., 2006).

Studies focusing on the analysis of electrode location and its correlation with good clinical outcomes suggested that an optimal stimulation point would be located in the lateral portion of the sensorimotor territory of GPi in proximity of lamina interna (Schönecker et al., 2015; Starr et al., 2006). Our results are in line with these findings showing that coordinates of such optimal stimulation sites are located within sensorimotor striatopallidal, subthalamopallidal, and pallidothalamic MPMs. It is worthy to note that, as expected, coordinates provided by both studies were located along the lateral border of striatopallidal and pallidothalamic MPMs while covered a more central position within subthalamopallidal MPM (see below).

Neumann et al. found that theta activity in the GPi is correlated to severity of dystonic symptoms and DBS in proximity of the peak of theta activity resulted in improved clinical outcomes. Interestingly, electrophysiological mapping of abnormal theta activity revealed that this peak is located in the posterior third of the nucleus (Neumann et al., 2017), which is likely to correspond to the sensorimotor area of the GPi. Indeed, our results showed that coordinates of both peak‐theta activity and average active lead location reported in (Neumann et al., 2017), resides within GPi sensorimotor territories reconstructed from striatopallidal, pallidothalamic, and subthalamopallidal pathways in the present study (Figure 7). In a multicenter study conducted on 105 implanted dystonic patients, group‐level aggregation of electrode locations and VTA were used to identify an anti‐dystonic sweet spot defined as the volume characterized by the highest probability of stimulation‐induced motor benefit. Such optimal stimulation site was located along the medial border of posteroventral GPi and in the adjacent subpallidal white matter (Reich et al., 2019), in close proximity of the borders of sensorimotor MPMs reconstructed herein. In the study by Okromelidze et al., conducted on 39 dystonic patients using VTA modeling, the highest correlation with clinical improvement as measured with UDRS was defined by a set of coordinates located in the ventrolateral GPi, along its border with the GPe (Okromelidze et al., 2020). Such coordinates slightly fell into the sensorimotor subthalamopallidal MPM sparing striatopallidal and pallidothalamic ones, even if being close to their lateral border. Such stimulation point is located near the medial border of GPe sensorimotor striatopallidal and subthalamopallidal MPMs, suggesting that the stimulation of white matter pathways passing through the GPe may also mediate DBS therapeutic effects as already hypothesized (Starr et al., 2006).

Finally, we also analyzed the Euclidean distances from each COG of sensorimotor maps obtained from different bundles of interest to each optimal stimulation site above described, in order to obtain a quantitative estimation of the spatial relations between connectivity parcels and such stimulation points.

The COG of subthalamopallidal‐derived sensorimotor parcellation was closest to coordinates of most of the optimal stimulation sites considered when compared to striatopallidal and pallidothalamic ones. If, on the one hand, these findings could suggest that an indirect, two‐stage, subthalamopallidal parcellation approach could be appropriate for targeting GPi in functional neurosurgery settings, more studies are needed to further reinforce this hypothesis. In addition, the STN is a very small structure and its connectivity patterns could be hard to study in common available clinical datasets that have usually lower spatial resolution, *b*‐values, and gradient directions compared to HCP data. The proximity of COG of subthalamopallidal sensorimotor maps to most of the optimal stimulation sites reported in literature may be interpreted considering the presence of theta activity not only in the GPi but also in the STN in dystonia patients. In this regard, Marsden and Obeso proposed that this abnormal subcortical theta synchronization may represent a “noisy signal” in the cortico‐basal ganglia‐thalamus circuit which is likely to affect the physiological motor network (Marsden & Obeso, 1994). However, the neuroimaging findings of the present work come from a sample of healthy subjects; therefore, they should be taken as a grain of salt and validated in future studies involving dystonia patients.

Finally, it should be kept in mind that COGs of tractography‐based connectivity maps do not necessarily correspond to optimal stimulation sites resulting in good clinical outcomes. In the present study, the COGs of each connectivity parcel have been chosen as a representative set of coordinates in order to provide a quantitative description of the spatial relations between established stimulation points and the connectivity parcels of the present study. Hence, an exact match is not necessarily to be expected.

Spatial relations between sensorimotor connectivity maps here proposed and available optimal stimulation sites could be also interpreted in light of models of dystonia as a network‐level disorder which ideally involves both the cortico‐basal ganglia‐thalamo‐cortical and cerebello‐thalamo‐cortical loops (Corp et al., 2019; Okromelidze et al., 2020). In a lesion network mapping study investigating functional connectivity of lesions leading to secondary dystonia, a negative connectivity to somatosensory cortex and a positive connectivity to cerebellum (vermis, cerebellar cortex, and dentate nucleus) resulted to be specific for cervical dystonia (Corp et al., 2019). In particular, the negative functional connectivity of somatosensory cortex was interpreted as a possible expression of a “missing inhibition” of such area in patients with dystonia and it may be hypothesized that DBS within sensorimotor region of GPi could restore such altered equilibrium (Okromelidze et al., 2020). In line with the model proposed by Corp and colleagues, a recent study showed that functional connectivity between VTA and primary sensorimotor regions, motor thalamus and cerebellum was positively correlated to clinical improvements. Hence, stimulation of thesensorimotor territory of GPi could exert an influence at a connectomic level inducing a “rewiring” which may result in the optimal connectivity profiles proposed by Okromelidze and colleagues. Moreover, both structural and functional connectivity profiles associated with UDRS improvement occurred with inferior parietal and lateral occipito‐temporal areas, corroborating the hypothesis according to which DBS may have modulatory effects on distributed brain networks (Horn, 2019). It can be hypothesized that the volume of GPi which is actually stimulated during DBS may exert an effect on surrounding and partially overlapping functional territories. In particular, our results show a valuable proximity between sensorimotor and “other” clusters, which include voxels that are supposed to be connected to the aforementioned parieto‐temporal‐occipital areas. However, further studies on dystonic patients are needed to support such hypothesis.

### 4.2 Methodological considerations

This work suffers from typical tractography limitations: this technique cannot distinguish polysynaptic from monosynaptic pathways and it does not allow inferences on polarity of connections preventing to differentiate afferent from efferent pathways (Jbabdi & Johansen‐Berg, 2011). Moreover, probabilistic tractography may often lead to “false positive” streamlines which do not correspond to ground truth (Maier‐Hein et al., 2017). Hence, in order to obtain more robust‐to‐noise and reliable results, we employed MSMT‐CSD, which reduces partial volume effects employing tissue‐type specific RFs (Jeurissen et al., 2014).

Connectivity clusters retrieved from CBP do not biologically imply that an effective parcellation exists within the structures considered. In other words, even if maps obtained from diffusion tractography may be defined as “connectivity modules” within parcellated structures, clear‐cut boundaries provided by parcellation techniques may not correspond to anatomical ground truth (Eickhoff et al., 2015). Moreover, CBP can be performed using different approaches, each one characterized by peculiar advantages and limitations. In the present work, we aimed at testing a specific hypothesis about spatial, topographical coherence in the basal ganglia that is derived from anatomical ground truth both in human and nonhuman primates and, therefore, we decided to employ a hypothesis‐driven approach for this aim (Karachi et al., 2002, 2005; Saga et al., 2017). Moreover, a previous study investigating the effects of different CBP approaches on GP segmentation revealed a good correspondence between hypothesis‐driven and data‐driven CBP methods (da Silva et al., 2017). Finally, the choice of a hard‐segmentation approach could represent an inherent limitation since it attributes voxels to the clusters exhibiting higher connectivity density (Behrens et al., 2003). Although widely used in literature (Cacciola, Milardi, Basile, et al., 2019; Cacciola, Milardi, Basile, et al., 2019; Middlebrooks, Tuna, Almeida, et al., 2018; Middlebrooks, Tuna, Grewal, et al., 2018), the use of such approach may introduce a bias selecting only the most connected voxels as part of each connectivity map therefore imposing a stricter parcellation in respect to the anatomical reality. This is particularly true when brain structures are thought to be composed of partially overlapping functional territories rather than well‐delineated subdivisions (Alkemade, Schnitzler, & Forstmann, 2015; Lambert et al., 2015; Plantinga et al., 2018).

However, the most common alternative to hard segmentation approach involves the selection of a connectivity threshold to filter out the voxels with lower connectivity values introducing an additional element of arbitrariness in the parcellation pipeline (Domin & Lotze, 2019; Johansen‐Berg et al., 2005; Tziortzi et al., 2014). Moreover, winner‐takes‐all parcellation appears to be better suited for clinical contexts where identifying the core locus of connectivity to a particular target region is likely to be more important than the connectivity territory as a whole (Clayden, Thomas, & Kraskov, 2019).

The choice of coordinates of electrode locations as optimal stimulation sites could also be considered as an inherent limitation. Indeed, it has been recently demonstrated that the mere contact location is a poor predictor of clinical outcome, with little to absent topographical difference between locations related with excellent outcomes and locations associated with poor outcomes (Reich et al., 2019). Even if VTA modeling has shown to be more consistent to identify sweet spots, such volumes are not easily available; thus, we opted to evaluate the possible translation of our results in clinical settings using available coordinates of optimal stimulation sites provided by different studies. Moreover, COG of MPMs are not expected to match optimal stimulation point within the connectivity map; indeed, the available coordinates related with good stimulation‐induced clinical outcomes showed various degree of distance with MPMs COG. However, this choice was guided by the need to provide a quantitative metric of the spatial relations between known stimulation points and the maps herein reconstructed by CBP. Indeed, despite heterogeneity in Euclidean distances, all stimulation points exhibited a plausible spatial relation in respect of striatopallidal, subthalamopallidal, and pallidothalamic maps.

Finally, the optimal stimulation points used in our paper derived from an average of patients coming from different studies. The process of transferring individual stimulation points to template coordinates and vice versa requires the use of nonlinear transformations which may further alter the precise localization of optimal stimulation points which is crucial in functional neurosurgery settings. We acknowledge that, when available, subject‐level information could lead to more clinically relevant results.

It is worth to mention that our connectivity maps derive from a sample of young (22–36 years) and healthy subjects; hence, our findings may not be generalizable to populations with different age. Thus, studies using larger and more representative samples are needed to confirm our results. In addition, our study is based on a healthy population and then we cannot rule out eventual alterations in individual connectivity profiles that may be related to the disease itself.

## 5 CONCLUSIONS

Taken together our results indicate that the topographical pattern of the functional territories of the GP is in line with the organizational pattern found in the striatum, STN and thalamus, thus further reinforcing the idea that the cortico‐basal ganglia‐thalamo‐cortical system is organized in parallel, spatially coherent and topographically organized pathways, that connect similar functional territories.

Moreover, a two‐stage CBP of GPi according to its connectivity patterns may be employed for a precise DBS targeting in functional neurosurgery for dystonia. Tractography‐derived sensorimotor maps may also represent a good candidate for therapeutic targeting, considering that DBS of sensorimotor GPi is likely to exert part of its therapeutic effect by modulating both local and distributed brain networks, likely inducing a “rewiring” of sensorimotor‐related connectivity.

In conclusion, the pallidal connectivity maps obtained in the present study may be employed during preoperative targeting in order to ameliorate clinical outcomes as well as to improve our current knowledge of side effects during postoperative evaluation. We hope that our results may shed new light on the macroscale, anatomical–functional organization of the basal ganglia circuitry.

Tractography‐based GP maps are publicly available at https://github.com/BrainMappingLab/GP_parcellation

## CONFLICT OF INTEREST

The authors declare no conflict of interest.

## ETHICS STATEMENT

Data were acquired by the Washington University, University of Minnesota, and Oxford University (WU‐Minn) HCP Consortium. The study was approved by the Washington University Institutional Review Board.

## PATIENTS CONSENT

Informed consent was obtained from all subjects.

## Supporting information


**Supplementary Figure S1** Multiple coronal sections depicting MPM derived from GPi (A) and GPe (B) according to striatopallidal tract, superimposed on the ICBM 2009b nonlinear asymmetric template. Connectivity maps were labeled as follows: limbic (red), associative (green), sensorimotor (blue), other (yellow).
**Supplementary Figure S2**. Multiple coronal sections depicting MPM derived from GPi (A) and GPe (B) according to subthalamopallidal pathway, superimposed on the ICBM 2009b nonlinear asymmetric template. Connectivity maps were labeled as follows: limbic (red), associative (green), sensorimotor (blue), other (yellow).
**Supplementary Figure S3**. Multiple coronal sections depicting MPM derived from GPi (A) according to pallidothalamic tract, superimposed on the ICBM 2009b nonlinear asymmetric template. Connectivity maps were labeled as follows: limbic (red), associative (green), sensorimotor (blue), other (yellow)

## Data Availability

One dataset of 100 unrelated healthy subjects was used to support the findings of this study. Data were provided by the Human Connectome Project, WU‐Minn Consortium (Principal Investigators: David Van Essen and Kamil Ugurbil; 1U54MH091657) and are publicly available at https://www.humanconnectome.org/study/hcp‐young‐adult/document/1200‐subjects‐data‐release. Tractography‐derived GP maps are publicly available at https://github.com/BrainMappingLab/GP_parcellation
