# Supplementary material for: Spatially coherent and topographically organized pathways of the human globus pallidus
Source: Hum Brain Mapp. 2020 Aug 5;41(16):4641–61. doi: 10.1002/hbm.25147 (PMC7555102; doi:10.1002/hbm.25147)
Supplement: Supplementary file 1 — Supplementary Figure S1 Multiple coronal sections depicting MPM derived from GPi (A) and GPe (B) according to striatopallidal tract, superimposed on the ICBM 2009b nonlinear asymmetric template. Connectivity maps were labeled as follows: limbic (red), associative (green), sensorimotor (blue), other (yellow). Supplementary Figure S2. Multiple coronal sections depicting MPM derived from GPi (A) and GPe (B) according to subthalamopallidal pathway, superimposed on the ICBM 2009b nonlinear asymmetric template. Connectivity maps were labeled as follows: limbic (red), associative (green), sensorimotor (blue), other (yellow). Supplementary Figure S3. Multiple coronal sections depicting MPM derived from GPi (A) according to pallidothalamic tract, superimposed on the ICBM 2009b nonlinear asymmetric template. Connectivity maps were labeled as follows: limbic (red), associative (green), sensorimotor (blue), other (yellow) [file HBM-41-4641-s001.docx]

**Supplementary Figures**


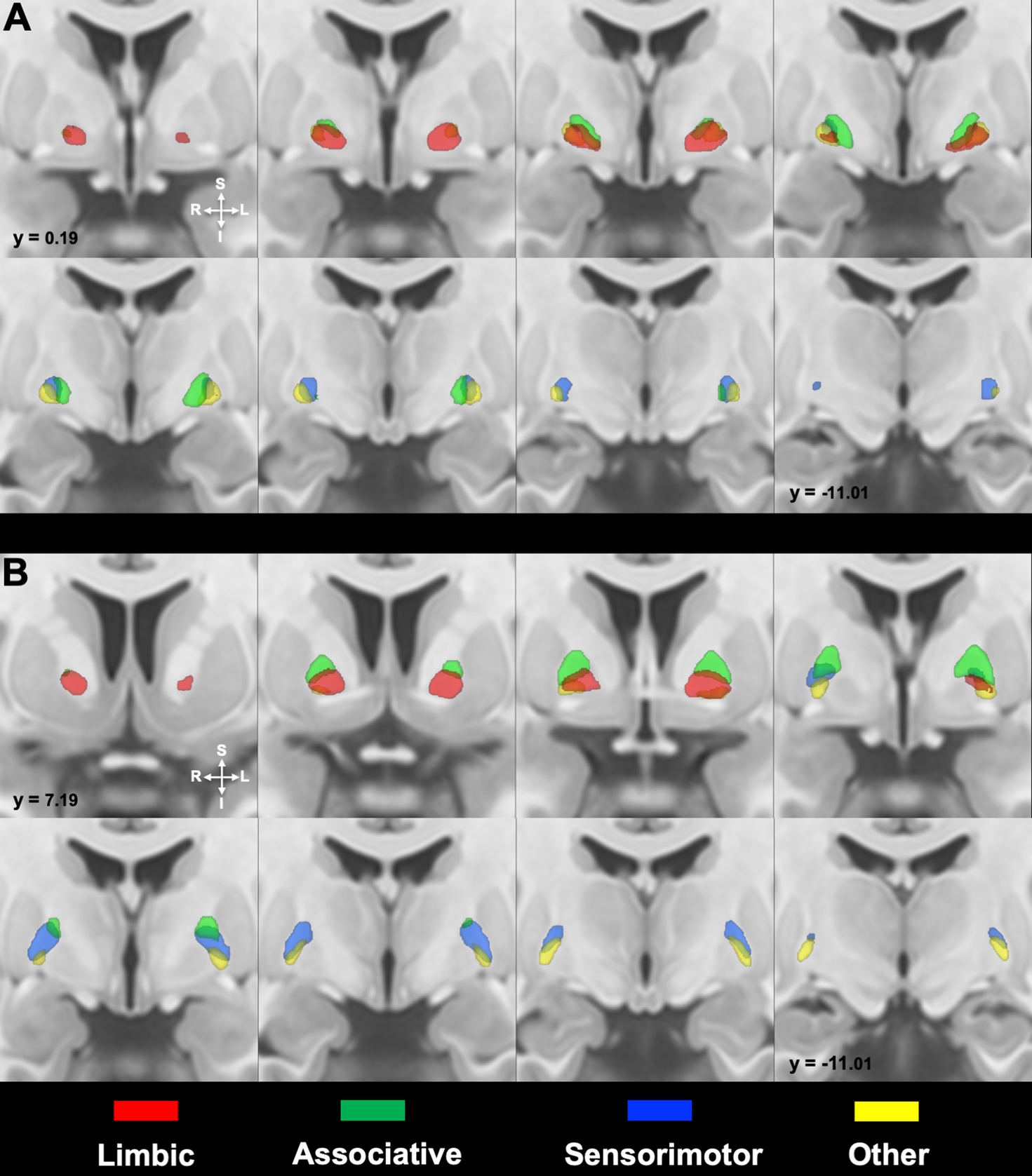


**Supplementary Figure 1.** Multiple coronal sections depicting MPM derived from GPi (A) and GPe (B) according to striatopallidal tract, superimposed on the ICBM 2009b nonlinear asymmetric template. Connectivity maps were labeled as follows: limbic (red), associative (green), sensorimotor (blue), other (yellow).


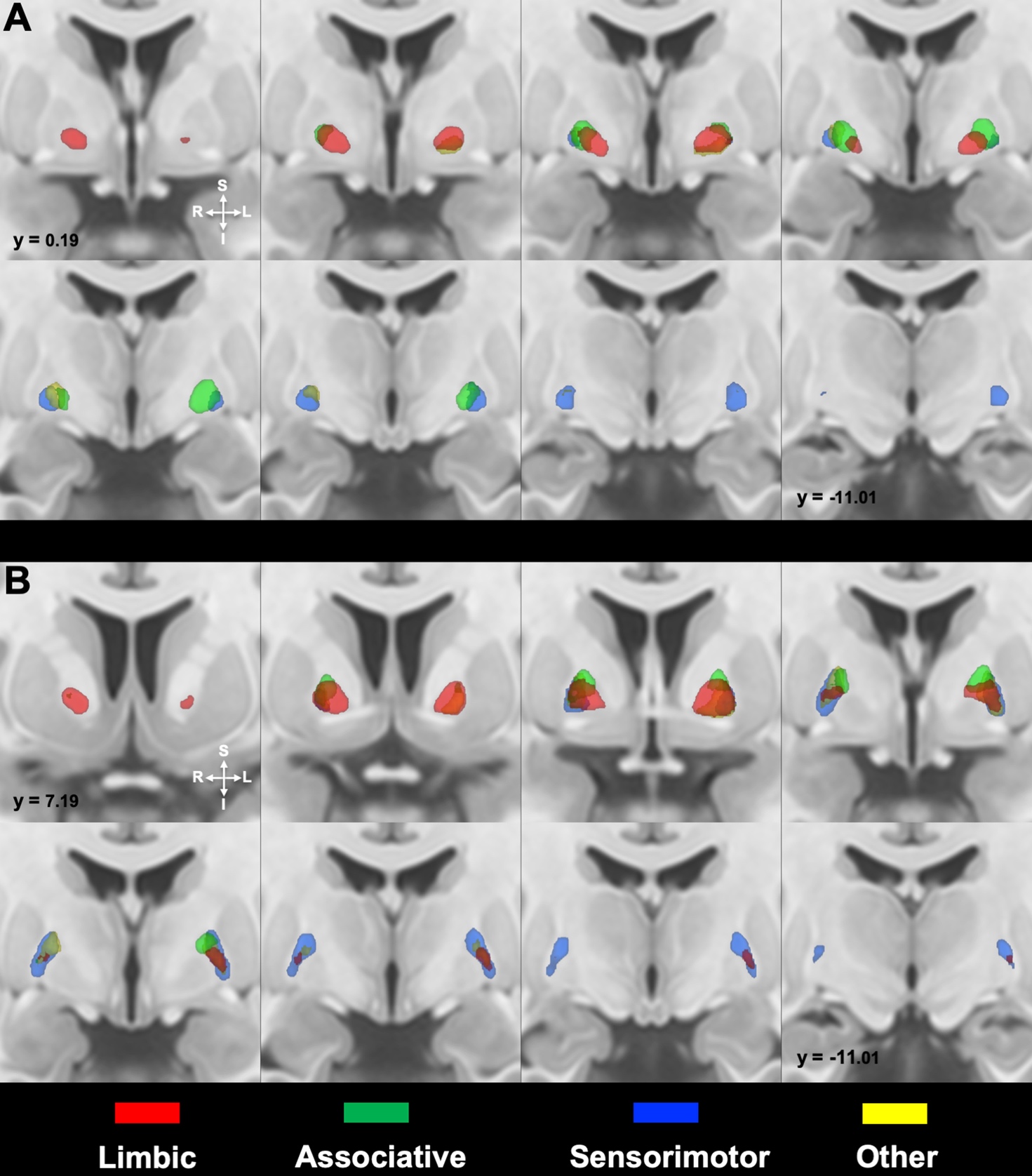


**Supplementary Figure 2.** Multiple coronal sections depicting MPM derived from GPi (A) and GPe (B) according to subthalamopallidal pathway, superimposed on the ICBM 2009b nonlinear asymmetric template. Connectivity maps were labeled as follows: limbic (red), associative (green), sensorimotor (blue), other (yellow).


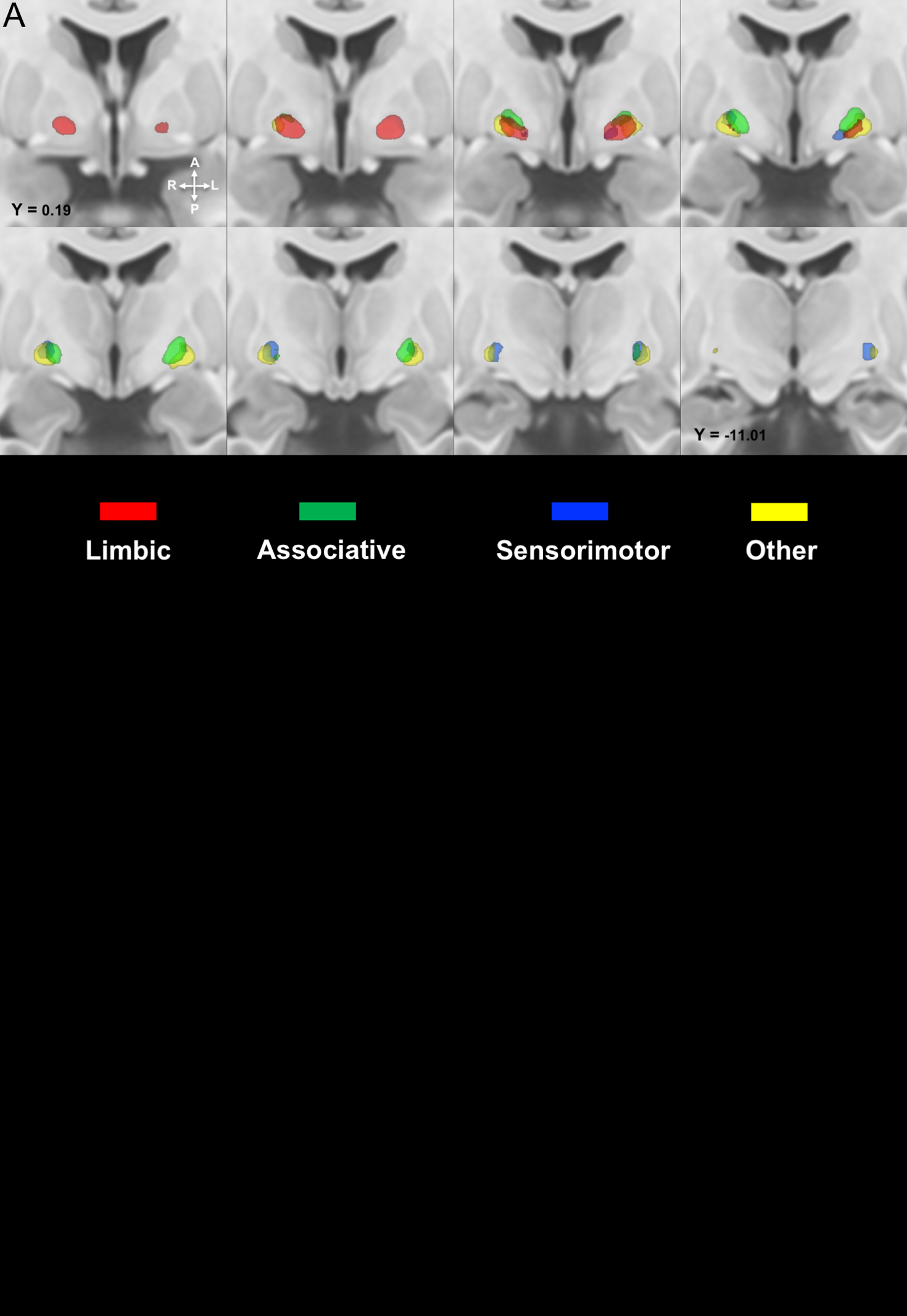
**Supplementary Figure 3.** Multiple coronal sections depicting MPM derived from GPi (A) according to pallidothalamic tract, superimposed on the ICBM 2009b nonlinear asymmetric template. Connectivity maps were labeled as follows: limbic (red), associative (green), sensorimotor (blue), other (yellow).
